# Dense Out-of-Distribution Detection by Robust Learning on Synthetic Negative Data

**DOI:** 10.3390/s24041248

**Published:** 2024-02-15

**Authors:** Matej Grcić, Petra Bevandić, Zoran Kalafatić, Siniša Šegvić

**Affiliations:** Faculty of Electrical Engineering and Computing, University of Zagreb, 10000 Zagreb, Croatia; matej.grcic@fer.hr (M.G.); petra.bevandic@fer.hr (P.B.); zoran.kalafatic@fer.hr (Z.K.)

**Keywords:** dense out-of-distribution detection, normalizing flows, semantic segmentation, autonomous driving, remote sensing

## Abstract

Standard machine learning is unable to accommodate inputs which do not belong to the training distribution. The resulting models often give rise to confident incorrect predictions which may lead to devastating consequences. This problem is especially demanding in the context of dense prediction since input images may be only partially anomalous. Previous work has addressed dense out-of-distribution detection by discriminative training with respect to off-the-shelf negative datasets. However, real negative data may lead to over-optimistic evaluation due to possible overlap with test anomalies. To this end, we extend this approach by generating synthetic negative patches along the border of the inlier manifold. We leverage a jointly trained normalizing flow due to a coverage-oriented learning objective and the capability to generate samples at different resolutions. We detect anomalies according to a principled information-theoretic criterion which can be consistently applied through training and inference. The resulting models set the new state of the art on benchmarks for out-of-distribution detection in road-driving scenes and remote sensing imagery despite minimal computational overhead.

## 1. Introduction

Image understanding involves recognizing objects and localizing them down to the pixel level [1]. In its basic form, the task is to classify each pixel into one of *K* predefined classes, which is also known as semantic segmentation [2]. Recent work improves perception quality through instance recognition [3], depth reconstruction [4], semantic forecasting [5], and competence in the open world [6].

Modern semantic segmentation approaches [2] are based on deep learning. A deep model for semantic segmentation maps the input RGB image x into the corresponding prediction y. Both the input and the predictions have the spatial resolution H×W, where *H* and *W* stand for the height and the width of the image. Typically, the model parameters θ are obtained by gradient optimization of a supervised discriminative objective. Recent approaches produce high-fidelity segmentations of large images in real time even when inferring on a modest graphical processing unit (GPU) [7]. However, standard learning is susceptible to overconfidence in incorrect predictions [8], which makes the model unusable in the presence of semantic outliers [9] and domain shift [10]. This poses a threat to models deployed in the real world [11,12].

We study the ability of deep models for natural image understanding to deal with out-of-distribution (OOD) input. We desire to correctly segment the scene while simultaneously detecting anomalous objects which are unlike any scenery from the training dataset [13]. Such capability is important in real-world applications like road driving [14,15] and remote sensing [16,17].

Previous approaches to dense OOD detection rely on Bayesian modeling [18], image resynthesis [14,19,20], recognition in the latent space [12], or auxiliary negative training data [21]. However, all these approaches have significant shortcomings. Bayesian approaches and image resynthesis require extraordinary computational resources that hamper development and make them unsuitable for real-time applications. Recognition in the latent space [12] may be sensitive to feature collapse [22,23] due to relying on pre-trained features. Training on auxiliary negative data may give rise to undesirable bias and over-optimistic evaluation. Moreover, appropriate negative data may be unavailable in some application areas, such as medical diagnostics [24] or remote sensing [16,25]. Our experiments suggest that synthetic negatives may help in such cases.

This work addresses dense out-of-distribution detection by encouraging the chosen standard dense prediction model to emit uniform predictions in outliers [26]. We propose to perform the training on mixed-content images [21], which we craft by pasting synthetic negatives into inlier training images. We learn to generate synthetic negatives by jointly optimizing high inlier likelihood and uniform discriminative prediction [26]. We argue that normalizing flows are better than generative adversarial networks (GANs) for the task at hand due to much better distribution coverage and more stable training. Also, normalizing flows can generate samples of variable spatial dimensions [27], which makes them suitable for mimicking anomalies of varying size.

This paper proposes five major improvements over our preliminary report [28]. First, we show that Jensen–Shannon divergence is a criterion of choice for robust joint learning in the presence of noisy synthetic negatives. We use the same criterion during inference as a score for OOD detection. Second, we propose to discourage overfitting the discriminative model to synthetic outliers through separate pre-training of the discriminative model and the generative flow. Third, we offer theoretical evidence for the advantage of our coverage-oriented synthetic negatives with respect to their adversarial counterparts. Fourth, we demonstrate the utility of synthetic outliers by performing experiments within the domain of remote sensing. These experiments show that off-the-shelf negative datasets, such as ImageNet, COCO, or Ade20k, do not represent a suitable source of negative content for all possible domains. Fifth, we show that training with synthetic negatives increases the separation between knowns and unknowns in the logit space, which makes our method a prominent component of future dense open-set recognition systems. We refer to the consolidated method as NFlowJS. NFlowJS achieves state-of-the-art performance on benchmarks for dense OOD detection in road driving scenes [11,12] and remote sensing images [16], despite not using auxiliary negative data [21], image resynthesis [14,19], and Bayesian modeling [18].

Our method has a very low overhead over the standard discriminative model, making it suitable for real-time applications.

## 2. Related Work

Several computer vision tasks require the detection of unknown visual concepts (Section 2.1). In practice, this often has to be integrated with some primary classification task (Section 2.2 and Section 2.3). Our method generates synthetic negatives with a normalizing flow due to outstanding distribution coverage and capability to work at arbitrary resolutions (Section 2.4).

### 2.1. Anomaly Detection

Anomaly detection, also known as novelty or out-of-distribution (OOD) detection, is a binary classification task which discriminates inliers from outliers [29,30]. In-distribution samples, also known as inliers, are generated by the same generative process as the training data. In contrast, anomalies are generated by a process which is disjoint from the training distribution [31]. Samples of anomalous data may or may not be present during the training [32,33]. The detection is typically carried out by thresholding some OOD score $s_{\delta }:{\left[0,1\right]}^{C\times H\times W}\to R$, which assigns a scalar score to each input image. Here, *C* stands for the number of channels, which is three in the case of RGB images. As before, *H* and *W* are the height and width, respectively.

Some works address OOD detection in isolation, as a distinct computer vision task [31,34,35,36,37,38,39]. Our work considers a different context where OOD detection is carried out alongside some discriminative dense prediction task, such as semantic segmentation.

### 2.2. Classification in the Presence of Outliers

OOD detection [40] can be implemented by extending standard classifiers. The resulting models can differentiate inliers while also detecting anomalous content. A widely used baseline expresses the OOD score for an image x directly from discriminative predictions as $s\left(x\right)=\max\mathrm{softmax}(f_{\theta }\left(x\right))$ [40], where $f_{\theta }$ computes dense logits based on the input image. Entropy-based detectors can deliver a similar performance [41,42]. Another line of work improves upon these baselines by pre-processing the input with anti-adversarial perturbations [32], which cause significant computational overhead. OOD detection has to deal with the fact that outliers and inliers may be indistinguishable in the feature space [23]. Feature collapse [22,43] can be alleviated by training on negative data, which can be sourced from real datasets [33,42] or generative models [26,28,44].

There are two prior approaches for replacing real negatives with synthetic ones [26,45]. A seminal approach [26] proposes cooperative training of a generative adversarial network and a standard classifier. The classifier loss requires uniform predictions in generated samples and, thus, encourages the generator to yield samples at the distribution border. This idea can be carried out without a separate generative model by leveraging Langevin sampling [45]. However, adapting these approaches for dense prediction is not straightforward. Similarly, synthetic outliers can be generated in the feature space by a generative model to pre-trained features [44,46]. However, our experiments indicate that this approach underperforms with respect to synthetic negative samples in input space.

Out-of-distribution detection becomes even more complicated in the case of object detection and dense prediction, where we have to deal with outlier objects in inlier scenes. These models strive to detect unknown hazards while correctly recognizing the rest of the scene [47,48,49]. A principled Bayesian approach to OOD detection attempts to estimate epistemic uncertainty [18]. However, the assumption that MC dropout corresponds to Bayesian model sampling may not be satisfied in practice. Another principled approach builds on likelihood estimation in feature space [12], which is vulnerable to feature collapse [22].

Another line of work resynthesizes the input scene by processing dense predictions with a conditional generative model [14,19,50]. Subsequently, anomalous pixels are detected in reconstructive fashion [30] by measuring the dissimilarity between the input and the resyntesized image. However, these approaches can detect anomalies only in front of simple backgrounds, such as roads. Also, resynthesis requires a significant computational budget, which limits applications. A related approach utilizes a parallel upsampling path for input reconstruction [15]. This improves the inference speed with respect to resynthesis approaches but still infers slower than our approach, while underperforming in cluttered scenes.

Several approaches train on mixed-content images obtained by pasting negative patches into positive training examples [19,21,51]. The negative dataset should be as broad as possible (e.g., ImageNet or ADE20k) in order to cover a large portion of the background distribution. The training can be implemented through a separate OOD head [21] or by requiring uniform prediction in negative pixels [51]. However, this kind of training results in biased models; test anomalies that are related to negative training data are going to give rise to above-average outlier detection performance. Furthermore, competition on popular benchmarks may gradually adapt negative training data to test anomalies, and, thus, lead to over-optimistic performance estimates. Our method avoids the bias of particular negative data by crafting problem-specific negative samples at the border of the inlier distribution.

### 2.3. Open-Set Recognition

Open-set recognition [52] discourages excessive generalization for known classes and attempts to distinguish them from the remaining visual content of the open world. This goal can be achieved by rejecting classification in input samples which do not belong to the known taxonomy [52,53,54,55]. The rejection mechanism is usually implemented by restricting the shape of the decision boundary [56]. This can be carried out by thresholding the distance from learned class prototypes in the embedding space [57,58]. The decision boundary can also be restricted by requiring a sufficiently large projection of the feature vector onto the closest class prototype [59]. This is also known as a max-logit detector, which can equally be used for OOD detection and open-set recognition [59,60].

Open-set recognition performance can be further improved by employing a stronger classifier [60] or training on negative data [61,62]. Unlike OOD detection approaches based on softmax, open-set recognition methods demonstrably bound open-space risk [52,63]. However, these approaches are still vulnerable to feature collapse [22]. We direct the reader to [64,65] for a broader overview of open-set recognition. Open-world approaches attempt to disentangle the detected unknown concepts towards new semantic classes. This can be performed in incremental [6,66] or low-shot [67,68,69] settings.

Although we mainly focus on OOD detection, our synthetic negatives could be considered as synthetic known unknowns [61,62]. Our experimental evaluation suggests that our synthetic negatives increase the separation between known and unknown data in feature space. This suggests that they may be helpful for open-set recognition [59,60].

### 2.4. Generative Models for Synthetic Negative Data

We briefly review generative approaches and discuss their suitability for generating synthetic negative training samples. Energy-based [70] and auto-regressive [71] approaches are unsuitable for this task due to slow sampling. Gaussian mixtures are capable of generating synthetic samples in the feature space [44]. Variational autoencoders (VAEs) [72] struggle with unstable training [73] and have to store both the encoder and the decoder in GPU memory. GANs [74] also require a roughly double amount of GPU memory since they have to backprop through the whole discriminator in order to train the generator. Moreover, the produced samples do not span the entire support of the training distribution [43]. In contrast, normalizing flows [27] offer both efficient sampling and outstanding distribution coverage [75].

Normalizing flows [27,76] model the likelihood as bijective mapping towards a predefined latent distribution p(z), typically a fully factorized Gaussian. Given a diffeomorphism $f_{\gamma }$ parametrized by γ, the likelihood is defined according to the change of variables formula:

(1) $$p_{\gamma }\left(x\right)=p\left(z\right)\left|\det\frac{\partial z}{\partial x}\right|,\;z=f_{\gamma }\left(x\right).$$

This setup can be further improved by introducing stochastic skip connections, which increase the efficiency of training and improve convergence speed [75].

A normalizing flow $f_{\gamma }$ can be sampled in two steps. First, we sample the latent distribution to obtain the factorized latent tensor z. Second, we recover the corresponding image through the inverse transformation $x=f_{\gamma }^{-1}\left(z\right)$. Both the latent representation and the generated image have the same dimensionality ($R^{C\times H\times W}\to {\left[0,1\right]}^{C\times H\times W}$). This property is useful for generating synthetic negatives since it allows to sample the same model on different spatial resolutions [27].

## 3. Dense OOD Detection with NFlowJS

We train dense OOD detection on mixed-content images obtained by pasting synthetic negatives into regular training images. We generate such negatives by a jointly trained normalizing flow (Section 3.1). We train our models to recognize outliers according to a robust information-theoretic criterion (Section 3.2), and use the same criterion as our OOD score during inference (Section 3.3). Finally, we present a theoretical analysis which advocates for training with synthetic negatives generated through likelihood maximization (Section 3.4).

### 3.1. Training with Synthetic Negative Data

We assemble a mixed-content image $x^{\prime }$ by sampling a randomly sized negative patch $x^{-}$ from a jointly trained normalizing flow $f_{\gamma }$, and pasting it atop the inlier image $x^{+}$:

(2) $$x^{\prime }=\left(1-s\right)\cdot x^{+}+\mathrm{pad}\left(x^{-},s\right)\;\mathrm{where}\;x^{-}=f_{\gamma }^{-1}\left(z\right).$$

The binary mask s identifies pixels of a pasted synthetic negative patch within the input image. As usual in normalizing flows, z is sampled from a factorized Gaussian and reshaped according to the desired spatial resolution. The negative patch $x^{-}$ is zero-padded in order to allow pasting by addition. The pasting location is selected randomly.

We train our discriminative model by minimizing the cross-entropy over inliers $\left(m^{ij}=0\right)$ and maximizing the prediction entropy in the pasted negatives $\left(m^{ij}=1\right)$ [26,33,42]:

(3) $$L_{\mathrm{disc}}\left(\theta ;\gamma \right)=\sum_{i,j}^{H,W}\left(m^{ij}-1\right)\cdot \ln p_{\theta }\left(y^{ij}|x^{\prime }\right)+\lambda \cdot m^{ij}\cdot L_{\mathrm{neg}}^{ij}\left(\theta ,\gamma \right).$$

**Figure 1:**
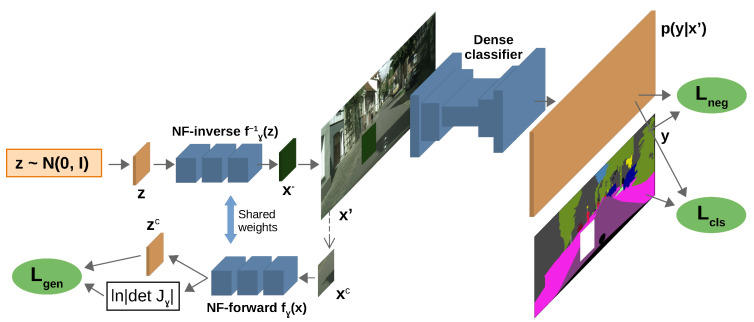
The proposed training setup. The normalizing flow generates the synthetic negative patch $x^{-}$, which we paste atop the raw inlier image. The resulting mixed-content image $x^{\prime }$ is fed to the dense classifier, which is trained to discriminate inlier pixels ($L_{\mathrm{cls}}$) and to produce uniform predictions in negative pixels ($L_{\mathrm{neg}}$). This formulation enables gradient flow from $L_{\mathrm{neg}}$ to the normalizing flow, while maximizing the likelihood of inlier patches ($L_{\mathrm{gen}}$).

Here, $p_{\theta }\left(y^{ij}|x^{\prime }\right)$ stands for the class posterior at ij pixel location and hyperparameter λ controls the influence of the loss $L_{\mathrm{neg}}$. We jointly train the normalizing flow alongside the primary discriminative model (cf. Figure 1) in order to satisfy two opposing criteria. First, the normalizing flow should maximize the likelihood of inlier patches. Second, the discriminative model should yield uniform distribution in generated pixels. The former criterion aligns the generative distribution with the inliers, while the latter pulls them apart. Such training encourages generation of synthetic samples at the boundary of the training distribution and incorporates outlier awareness within the primary discriminative model [26]. The total loss applied to the generative model equals to:

(4) $$L_{\mathrm{gen}}\left(\gamma ;\theta \right)=L_{\mathrm{nll}}\left(\gamma \right)+\lambda \sum_{i,j}^{H,W}m^{ij}\cdot L_{\mathrm{neg}}^{ij}\left(\theta ,\gamma \right).$$

$L_{\mathrm{nll}}$ is the negative log-likelihood of the inlier patch which is replaced with the synthetic sample. Formally, we have $L_{\mathrm{nll}}\left(\gamma \right)=-\ln p_{\gamma }\left(x^{c}\right)$, where $p_{\gamma }$ is defined in (1) and $x^{c}$ is a random crop of the given input image, as shown in Figure 1. We scrutinize $L_{\mathrm{neg}}$ in the following section. The end-to-end training learns both θ and γ simultaneously by optimizing the following loss:

(5) $$L\left(\theta ,\gamma \right)=L_{\mathrm{disc}}\left(\theta ;\gamma \right)+L_{\mathrm{gen}}\left(\gamma ;\theta \right).$$

**Figure 2:**
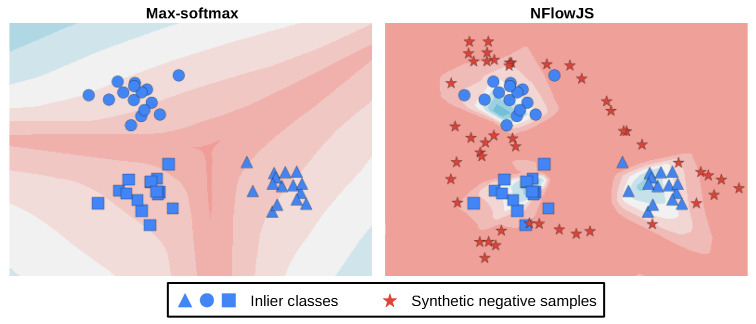
Softmax-activated discriminative models do not bound the input-space volume with confident predictions (blue region, **left**). We address this issue by learning a generative normalizing flow for a “negative” distribution that encompasses the training manifold (red stars, **right**). Training the discriminative model to predict high entropy in the generated synthetic negative samples decreases the confidence outside the inlier manifold (red region, **right**).

Given enough training data and appropriate capacity, our synthetic negatives will encompass the inlier manifold. Consequently, our method stands a fair chance to detect visual anomalies that had not been seen during training due to being closer to the synthetic negatives than to the inliers. Figure 2 shows this on a 2D toy example. The red color corresponds to higher values of the OOD score. The left plot presents the max-softmax baseline [40], which assigns a high OOD score only at the border between the inlier classes. The right plot corresponds to our setup, which discourages low OOD scores outside the inlier manifold. Synthetic negatives are denoted with red stars, while inlier classes are colored in blue.

### 3.2. Loss in Synthetic Negative Pixels

The loss $L_{\mathrm{neg}}$ has often been designed as KL-divergence between the uniform distribution and the model’s predictive distribution [26,40,42]. However, our generative model is also subjected to the $L_{\mathrm{nll}}$ loss. Hence, the generated samples occasionally contain parts very similar to chunks of inlier scenes, which lead to confident predictions into a known class. Unfortunately, such predictions lead to unbounded penalization by KL divergence and can disturb the classifier, which is also affected by $L_{\mathrm{neg}}$. If $L_{\mathrm{neg}}$ overrides $L_{\mathrm{disc}}$ in such pixels, then the classifier may assign high uncertainty in the inliers. In that case, the incidence of false-positive anomalies would severely increase. We address this problem by searching for a more robust formulation of $L_{\mathrm{neg}}$.

**Figure 3:**
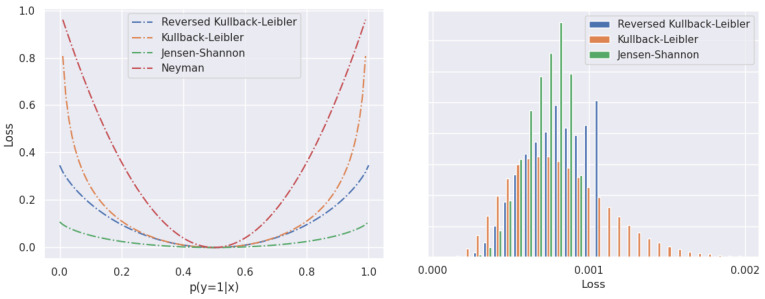
(**Left**) f-divergences towards the uniform distribution in a two-class setup. Jensen–Shannon offers the most robust response. (**Right**) Histograms of $\lambda L_{\mathrm{neg}}$ in synthetic negatives at the beginning of joint fine-tuning. The modulation factors λ have been separately validated for each of the three choices of $L_{\mathrm{neg}}$. The Jensen–Shannon divergence produces a more uniform learning signal than other f-divergences and avoids extremely high values of $L_{\mathrm{neg}}$.

The left part of Figure 3 plots several f-divergences in the two-class setup. We observe that the Jensen–Shannon divergence mildly penalizes high-confidence predictions, which makes it a suitable candidate for a robust loss. Such behavior promotes graceful performance degradation in cases of errors of the generative model. The right part of Figure 3 visualizes a histogram of per-pixel loss while fine-tuning our model on road-driving images. The figure shows that the histogram of JS divergence has fewer high-loss pixels than the other f-divergence candidates. Long tails of the KL divergences (forward and reverse) indicate a very high loss in pixels that resemble inliers. As hinted before, these pixels give rise to very high gradients with respect to the parameters of the discriminative model. These gradients may override the impact of the standard discriminative loss $L_{\mathrm{disc}}$, and lead to high-entropy discriminative predictions that disrupt our anomaly score and lead to false-positive predictions.

Consequently, we formulate $L_{\mathrm{neg}}$ in terms of the JS divergence between the uniform distribution (U) over classes and the softmax output:

(6) $$L_{\mathrm{neg}}^{ij}\left(\theta ,\gamma \right)=\mathrm{JS}\left(U,p_{\theta }\left(y^{ij}|x^{\prime }\right)\right)$$

### 3.3. Outlier-Aware Inference with Divergence-Based Scoring

**Figure 4:**
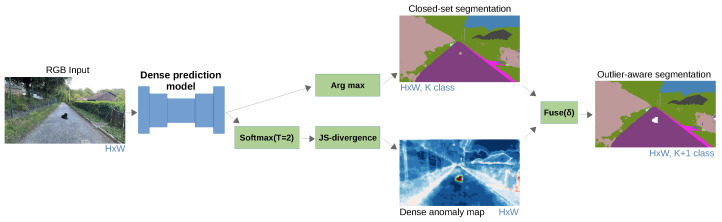
Dense outlier-aware inference. We infer dense logits with a closed-set model. We recover the dense OOD map according to our divergence-based score (JSD). Closed-set predictions are overridden in the outlier-aware output wherever the OOD score exceeds the threshold δ.

Figure 4 summarizes inference according to the proposed method for outlier-aware semantic segmentation. The input image is fed into the discriminative model. The produced logits are fed into two branches. The top branch delivers closed-set predictions through arg-max. The bottom branch recovers the dense OOD map through temperature scaling, softmax, and JS divergence with respect to the uniform distribution. Our dense OOD score at every pixel i,j reflects the $L_{\mathrm{neg}}$ loss (6):

(7) $$s^{ij}\left(x\right)=\mathrm{JS}\left(U,\mathrm{softmax}\left(l^{ij}/T\right)\right).$$

U stands for uniform distribution over inlier classes, l represents logits, while *T* is a temperature hyperparameter. The two branches are fused into the final outlier-aware segmentation map. The OOD map overrides the closed-set prediction wherever the OOD score exceeds a dataset-wide threshold.

Temperature scaling [8] reduces the relative OOD score of distributions with two dominant logits as opposed to distributions with homogeneous non-maximum logits. This discourages false-positive OOD responses at semantic borders. We use the same temperature T = 2 in all experimental comparisons with respect to previous methods. Note that our inference is very fast since we use our generative model only to simulate anomalies during training. This is different from image resynthesis [14] and embedding density [12], where the generative model has to be used during inference. Next, we compare the distributional coverage of synthetic negatives generated by the normalizing flow with respect to their GAN-generated counterparts.

### 3.4. Coverage-Oriented Generation of Synthetic Negatives

We provide a theoretical argument that our synthetic negatives provide a better distribution coverage than their GAN counterparts [26]. Our argument proceeds by analyzing the gradient of the joint loss with respect to the generator of synthetic negatives for both approaches. For brevity, we consider image-wide classification and omit the loss modulation hyperparameters.

Adversarial outlier-aware learning [26] jointly optimizes the zero-sum game between the generator $G_{\psi }$ and the discriminator $D_{\phi }$, the closed-set classification $P_{\theta }$, and the confidence objective that enforces uncertain classification in the negative data points [26]:

(8) $$\begin{array}{c} L_{\mathrm{adv}}\left(\phi ,\psi ,\theta \right)=\int p^{*}\left(x\right)\ln D_{\phi }\left(x\right)\;dx+\int p_{G_{\psi }}\left(x\right)\ln\left(1-D_{\phi }\left(x\right)\right)\;dx\\ \;\;\;\;\;\;\;\;\;\;\;\;\;\;\;\;\;\;\;\;\;\;\;\;\;\;\;\;\;\;\;\;\;\;\;\;\;\;\;\;\;\;\;\;\;\;\;\;\;\;\;\;\;-\int \sum_{y}p^{*}\left(y,x\right)\ln P_{\theta }\left(y|x\right)\;dx+\int p_{G_{\psi }}\left(x\right)F\left(P_{\theta },U\right)\;dx. \end{array}$$

We denote the true data distribution as $p^{*}$, *y* is the class, and ϕ,ψ, and θ are learnable parameters, while F corresponds to the chosen f-divergence. The gradient of the joint loss (8) with respect to the generator parameters ψ vanishes in the first and the third term. The remaining terms enforce that the generated samples fool the discriminator and yield high-entropy closed-set predictions:

(9) $$\frac{\partial L_{\mathrm{adv}}\left(\phi ,\psi ,\theta \right)}{\partial \psi }=\frac{\partial }{\partial \psi }\int p_{G_{\psi }}\left(x\right)\ln\left(1-D_{\phi }\left(x\right)\right)\;dx+\frac{\partial }{\partial \psi }\int p_{G_{\psi }}\left(x\right)F\left(P_{\theta },U\right)\;dx.$$

However, fooling the discriminator does not imply distributional coverage. In fact, the adversarial objective may cause mode collapse [77], which is detrimental to sample variability.

Our joint learning objective (5) optimizes the likelihood of inlier samples, the closed-set classification loss, and low confidence in synthetic negatives:

(10) $$L\left(\theta ,\gamma \right)=-\int p^{*}\left(x\right)\ln p_{\gamma }\left(x\right)\;dx-\int \sum_{y}p^{*}\left(y,x\right)\ln P_{\theta }\left(y|x\right)\;dx+\int p_{\gamma }\left(x\right)F\left(P_{\theta },U\right)\;dx.$$

The gradient of the loss (10) with respect to the normalizing flow parameters γ vanishes in the second term. The remaining terms enforce that the generated samples cover all modes of $p^{*}$ and, as before, yield high-entropy discriminative predictions:

(11) $$\frac{\partial L(\theta ,\gamma )}{\partial \gamma }=-\frac{\partial }{\partial \gamma }\int p^{*}\left(x\right)\ln p_{\gamma }\left(x\right)\;dx+\frac{\partial }{\partial \gamma }\int p_{\gamma }\left(x\right)F\left(P_{\theta },U\right)\;dx.$$

The resulting gradient entices the generative model to produce samples along the border of the inlier distribution. Hence, we say that our synthetic negatives are coverage-oriented. The presented analysis holds for any generative model that optimizes the density of the training data. The experimental evaluations in Section 5 provide empirical confirmation for the advantages of synthetic negatives generated by the normalizing flow (cf. the table in Section 6.2).

## 4. Experimental Setup

This section describes our experimental setup for dense out-of-distribution detection. We review the datasets, introduce performance metrics, and describe the training details.

### 4.1. Benchmarks and Datasets

**Figure 5:**
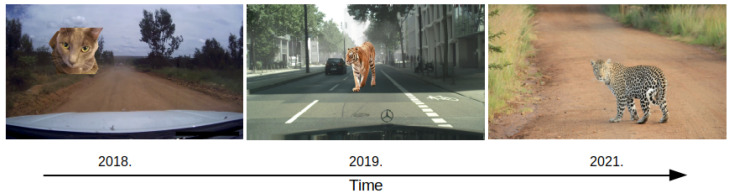
Development of dense OOD detection in road driving through time. Early work pastes objects at random locations [79]. This was improved by carefully choosing pasting locations and post-processing [12]. Contemporary outliers match the environment from the real-world scenes [11].

Benchmarks for dense OOD detection in road-driving scenes have experienced substantial progress in recent years (cf. Figure 5). In parallel, significant effort has been invested into artificial datasets by leveraging simulated environments [59,78]. Similarly, remote-sensing segmentation datasets have grown both in size in complexity [16].

We test our method on WD-Pascal [79], which allows for evaluating outlier detection in demanding conditions. However, the random pasting policy disturbs the scene layout, as shown in Figure 5 (left). We test our method on Fishyscapes [12], which consists of two datasets: FS LostAndFound and FS Static. FS LostAndFound is a small subset of original LostAndFound [80], which contains small objects on the roadway (e.g., toys, boxes, or car parts that could fall off). FS Static contains Cityscapes validation images overlaid with Pascal VOC objects. The objects are positioned according to the perspective and further post-processed to obtain smoother OOD injection, as shown in Figure 5 (center). Also, we test on SegmentMeIfYouCan (SMIYC) [11], which consists of three datasets: AnomalyTrack, ObstacleTrack, and LostAndFound-noKnown. AnomalyTrack provides large anomalous objects, which are fully aligned with the environment. For instance, they have a leopard in the middle of a dirt road, as shown in Figure 5 (right). LostAndFound-NoKnown tests the detection of small hazardous objects (e.g., boxes, toys, car parts, etc.) in urban scenes. Finally, ObstacleTrack tests the detection of small objects on various road types. ObstacleTrack and LostAndFound measure OOD detection performance solely on the driving surface, while AnomalyTrack considers the detection across the whole image. Consequently, SMIYC provides a solid notion of OOD segmentation performance of a model deployed in the wild. The last test dataset is StreetHazards [59], which is simulated with the CARLA game engine. We use StreetHazards for measuring outlier-aware segmentation according to open-mIoU [81].

We test our method on remote sensing images from BSB [16], which captures aerial images of Brasilia. It contains 3400 labeled images of 512 × 512 pixels. The official split designates 3000 train, 200 validation, and 200 test images. The labels include three stuff classes (street, permeable area, and lake) and 11 thing classes (e.g., swimming pool, vehicle, sports court). We extract boat and harbour into the OOD test set. The resulting BSB-OOD dataset contains 2840 training images with 12 inlier classes, while the OOD test set contains 184 images. This setup is similar to [28,59,82] that also select a subset of classes as OOD samples. Note that there are other remote sensing datasets, such as Vaihingen and Potsdam from the International Society for Photogrammetry and Remote Sensing (ISPRS). However, these datasets have fewer labels and an order of magnitude fewer images. Also, the So2Sat LCZ42 dataset [83] contains only small-resolution images and image-level labels. Hence, we opt for a larger dataset and better performance estimates.

### 4.2. Metrics

We measure OOD segmentation performance using the average precision (AP) [1], the false-positive rate at a true-positive rate of 95% (${\mathrm{FPR}}_{95}$) [40], and the area under the receiver operating characteristic curve (AUROC). AP is well suited for measuring OOD detection performance since it emphasizes the minority class [11,12,84]. A perfect OOD detector would have AP equal to one. Likewise, ${\mathrm{FPR}}_{95}$ is significant for real-world applications since high false-positive rates would require a large number of human interventions in practical deployments and, therefore, severely diminish the practical value of an autonomous system. We measure outlier-aware segmentation performance by open-mIoU [81]. Open-mIoU penalizes outliers being recognized as inliers and inliers being wrongly detected as outliers. Compared to mIoU over K + 1 classes, open-mIoU does not count true-positive outlier predictions and averages over K instead of K + 1 classes. The Open-mIoU performance of an outlier-aware segmentation model with ideal OOD detection would be equal to the closed-set mIoU of the same model. Hence, the difference between the two metrics quantifies the performance gap caused by the presence of outliers [81].

### 4.3. Implementation Details

All our models are based on Ladder DenseNet-121 (LDN-121) due to memory efficiency and fast experimentation [85]. However, our framework can accommodate any other dense prediction architecture. All our experiments consist of two training stages. In both stages, we utilize Cityscapes [86], Vistas [87], and Wilddash 2 [9]. These three datasets contain 25,231 images. The images are resized to 1024 pixels (shorter edge), randomly flipped with the probability of 0.5, randomly resized in the interval [0.5,2], and randomly cropped to 768×768 pixels. We optimize our models with Adam. In the first stage, we train for 25 epochs without synthetic negatives. We use a batch size of 16 as validated in previous work [85]. The starting learning rate is set to $10^{-4}$ for the feature extractor and $4\times 10^{-4}$ for the upsampling path. The learning rate is annealed according to a cosine schedule to the minimal value of $10^{-7}$, which would have been reached in the 50th epoch.

In the second stage, we train for 15 epochs on mixed-content images (cf. Section 3.1). In this stage, we use a batch size of 12 due to limited GPU memory. We did not use gradient accumulation due to the batch normalization layers. Instead, we opted for gradient checkpointing [85,88,89]. The initial learning rate is set to $1\times 10^{-5}$ for the upsampling path and $2.5\times 10^{-6}$ for the backbone. Once more the learning rate is decayed according to the cosine schedule to the value of $10^{-7}$. We set the hyperparameter λ to $3\times 10^{-2}$.

This value is chosen so that the closed-set segmentation performance is not reduced.

We generate rectangular synthetic samples with dimensions from U(16,216) by leveraging DenseFlow-25-6 [75]. The flow is pre-trained on random 64×64 crops from Vistas. We train the flow with the Adamax optimizer with the learning rate set to $10^{-6}$. In the case of WD-Pascal, we train our model only on Vistas in order to achieve a fair comparison with the previous work [21]. In the case of StreetHazards, we train on the corresponding train subset for 80 epochs on inlier images and 40 epochs on mixed-content images. In the case of Fishyscapes, we train exclusively on Cityscapes. We train for 150 epochs during stage 1 (inliers) and 50 epochs during stage 2 (mixed content). In the case of the BSB-OOD dataset, we train LDN-121 for 150 epochs with a batch size of 16 on inlier images and then fine-tune on mixed-content images for 40 epochs. We sample synthetic negatives with dimensions from U(16,64). The flow was pre-trained on 32×32 random inlier crops of BSB-OOD images for 2k epochs with a batch size of 256. All other hyperparameters are kept constant across all experiments. Each experiment lasts for approximately 38 h on a single GPU.

## 5. Experimental Evaluation

We evaluate OOD detection performance on road-driving scenes and aerial images. Road-driving experiments suggest that our synthetic negatives can deliver comparable performance to real negatives (Section 5.1). Our synthetic negatives become a method of choice in setups with a large domain between the inliers and negative datasets (Section 5.2).

We compare our performance with respect to contemporary methods which do not require the negative dataset and image resynthesis. We list all methods in our tables, so we can discuss our method in a broader context. We also analyze the sensitivity of our method with respect to the distance of the OOD object from the camera. Finally, we measure the computational overhead of our method with respect to the baseline and visualize our synthetic samples.

### 5.1. Dense Out-of-Distribution Detection in Road-Driving Scenes

Table 1 presents the performance on WD-Pascal averaged over 50 runs [21]. All the methods have been trained on the Vistas dataset and achieve similar mIoU performance. The column “Aux Data” indicates whether the method trains on real negative data. We choose ADE20k for this purpose since it offers instance-level ground truth. The bottom section compares our method with early approaches: MC dropout [18], ODIN [32], and max-softmax [40]. These approaches are not competitive with the current state-of-the-art. The top section shows that training with auxiliary negative data can significantly improve performance. However, our method closes the performance gap. It outperforms all other methods in ${\mathrm{FPR}}_{95}$ and AUROC metrics while achieving competitive AP.

Table 2 presents a performance evaluation of SMIYC [11] and Fishyscapes [12]. Our method outperforms all previous methods on AnomalyTrack, ObstacleTrack, as well as LAF-noKnown. We achieve such results despite refraining from image resynthesis [14,19,20], partial image reconstruction [15], or training on real negative images [12]. Our method achieves very low ${\mathrm{FPR}}_{95}$ (less than 1%) on ObstacleTrack and LostAndFound-noKnown. This is especially important for real-world applications, where a high incidence of false-positive anomalies may make OOD detection useless. Note that ObstacleTrack includes small obstacles in front of a variety of road surfaces, which makes it extremely hard not to misclassify road parts as anomalies. Moreover, this dataset includes low-visibility images captured at dusk and other challenging evaluation setups. Our synthetic negative data also achieve competitive performance on FS LostAndFound. Our method outperforms others in terms of ${\mathrm{FPR}}_{95}$, while achieving the second best AP. We slightly underperform only with respect to SynBoost, which trains on real negative data and precludes real-time inference due to image resynthesis. In the case of the FS Static dataset, our method achieves the best ${\mathrm{FPR}}_{95}$ and the second best AP among the methods which do not train on auxiliary data.

We also applied our method to a pre-trained third-party closed-set model and submitted the results to the Fishyscapes benchmark. We chose a popular DeepLabV3+ model which achieves high performance due to training on unlabeled video data [90]. This choice promotes fair comparison, since the same model has also been used in several other benchmark submissions [15,91]. Please note that we use parameters which have not been trained on Cityscapes val in order to allow fair evaluation on FS Static. The corresponding dense OOD detection model achieves 43.7 AP and 8.6 ${\mathrm{FPR}}_{95}$ on FS LAF, 54.7 AP, and 10.0 ${\mathrm{FPR}}_{95}$ on FS Static, while having 80.7 mIoU on Cityscapes val. We do not show these results in Table 2 in order to keep the same model across all assays. This result clearly shows that our method can also be applied to third-party models and deliver strong results.

**Table 2:** Dense out-of-distribution detection performance on SegmentMeIfYouCan and Fishyscapes.

Method			SegmentMeIfYouCan [11]						Fishyscapes [12]				
	Aux	Img	Anomalies		Obstacles		LAF-nK		FS LAF		FS Static		CS val
	Data	Rsyn.	AP	${\mathrm{FPR}}_{95}$	AP	${\mathrm{FPR}}_{95}$	AP	${\mathrm{FPR}}_{95}$	AP	${\mathrm{FPR}}_{95}$	AP	${\mathrm{FPR}}_{95}$	$\bar{\mathrm{IoU}}$
SynBoost [19]	✓	✓	56.4	61.9	71.3	3.2	81.7	4.6	43.2	15.8	72.6	18.8	81.4
Prior Entropy [92]	✓	✗	-	-	-	-	-	-	34.3	47.4	31.3	84.6	70.5
OOD Head [21]	✓	✗	-	-	-	-	-	-	31.3	19.0	96.8	0.3	79.6
Void Classifier [12]	✓	✗	36.6	63.5	10.4	41.5	4.8	47.0	10.3	22.1	45.0	19.4	70.4
Image Resyn. [14]	✗	✓	52.3	25.9	37.7	4.7	57.1	8.8	5.7	48.1	29.6	27.1	81.4
Road Inpaint. [20]	✗	✓	-	-	54.1	47.1	82.9	35.8	-	-	-	-	-
Max softmax [40]	✗	✗	28.0	72.1	15.7	16.6	30.1	33.2	1.8	44.9	12.9	39.8	80.3
MC Dropout [18]	✗	✗	28.9	69.5	4.9	50.3	36.8	35.6	-	-	-	-	-
ODIN [32]	✗	✗	33.1	71.7	22.1	15.3	52.9	30.0	-	-	-	-	-
SML [91]	✗	✗	-	-	-	-	-	-	-	31.7	21.9	52.1	20.5
Embed. Dens. [12]	✗	✗	37.5	70.8	0.8	46.4	61.7	10.4	4.3	47.2	62.1	17.4	80.3
JSRNet [15]	✗	✗	33.6	43.9	28.1	28.9	74.2	6.6	-	-	-	-	-
NFlowJS (ours)	✗	✗	56.9	34.7	85.5	0.4	89.3	0.7	39.4	9.0	52.1	15.4	77.4

**Figure 6:**
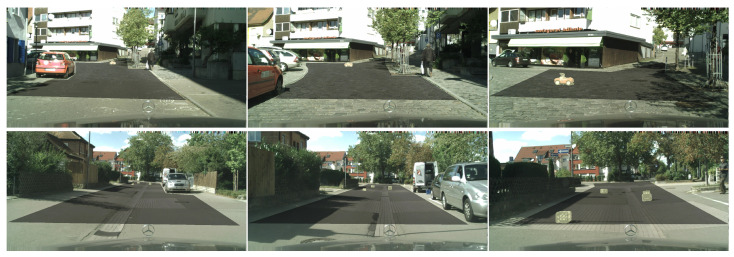
OOD detection on LostAndFound dataset. Our method can detect obstacles at different distances from the camera (**top**) as well as multiple obstacles in one image (**bottom**). The road ground truth is designated in grey and the predicted OOD in yellow. Zoom in to see the distant obstacles.

Figure 6 shows qualitative performance on two sequences of images from SMIYC LostAndFound. The rad-surface ground truth is designated in grey while the detected obstacles are in yellow. The top sequence contains obstacles which change position through time. The bottom sequence contains multiple anomalous objects. Our method succeeds in detecting a toy car and cardboard boxes even though no such objects were present during the training. The leftmost image contains distant obstacles, so please zoom in for better visibility.

Table 3 shows OOD detection and outlier-aware semantic segmentation on StreetHazards. We produce outlier-aware semantic predictions by correcting closed-set predictions with our dense OOD map (Section 3.3). We validate the OOD threshold in order to achieve TPR = 95% [81] and measure performance according to mIoU over K + 1 classes as well as with open-mIoU [81]. To the best of our knowledge, our method outperforms all previous work. In particular, our method is better than methods which utilize auxiliary negative datasets [21,33,93] and the method based on image resynthesis [50]. We note that there is still a significant performance degradation in the presence of outliers. The closed-set performance is more than 65% mIoU, while the outlier-aware performance peaks at 45%. Future research should strive to close this gap to provide safer segmentation in the wild.

We incorporated [32,33,93,96] into our codebase according to official implementations. For the energy fine-tuning, we conducted hyperparameter search as suggested in [93]: $m_{in}\in \left\{-15,-23,-27\right\}$ and $m_{out}\in \left\{-5,-7\right\}$. The optimal values for the dense setup are $m_{in}=-15$ and $m_{out}=-5$. We validated ReAct [96] for c∈{0.9,0.95,0.99}. The best results are obtained with c=0.99.

**Figure 7:**
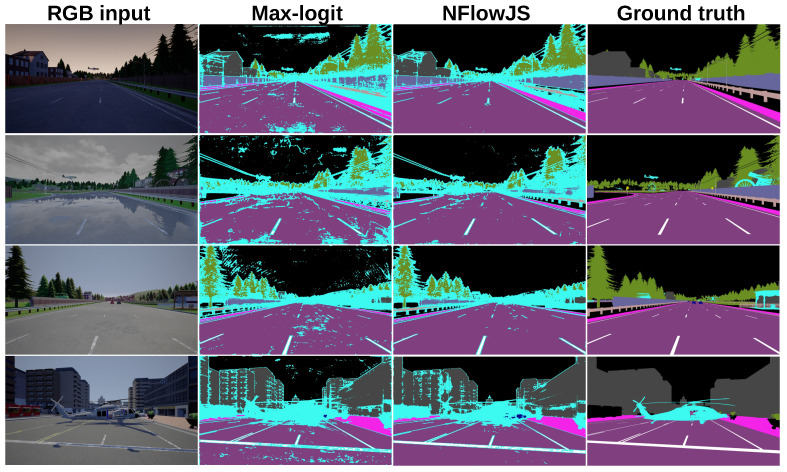
Outlier-aware segmentation on StreetHazards. The detected outliers are marked with cyan. Our method reduces the number of false-positives over the max-logit baseline.

Figure 7 compares the outlier-aware semantic segmentation performance of the proposed method with respect to the max-logit baseline [59] on StreetHazards. Anomalous pixels are designated in cyan. Our method reduces the number of false-positive anomalies. However, safe and accurate outlier-aware segmentation is still an open problem.

### 5.2. Dense Out-of-Distribution Detection in Remote Sensing

We compare our method with standard baselines for OOD detection [33,40,93], as well as with methods specifically developed for OOD detection in remote sensing imagery [17,25]. Table 4 shows the performance on the BSB-aerial-OOD dataset [16]. Some methods train on real negative data (cf. Aux data). The top section presents several OOD detection baselines. We observe that training with real negative samples outperforms the MSP baseline [40] but underperforms with respect to our synthetic samples. This is not surprising since the pasted negative instances involve a different camera perspective than aerial imagery. The middle section presents methods that are explicitly designed for aerial images. Morph-OpenPixel (MOP) [17] erodes the prediction confidence at object boundaries with morphological filtering. Morphological filtering improves ${\mathrm{FPR}}_{95}$ but impairs AP with respect to the MSP baseline. ${\mathrm{DPN}}^{-}$ [25] achieves runner-up AUROC and ${\mathrm{FPR}}_{95}$ performance. The bottom part shows the performance of our model. JSDiv is the same as NFlowJS except that it uses negatives from ADE20k instead of synthetic ones. NFlowJS generates dataset-specific negatives along the border between the known and the unknown. NFlowJS outperforms methods which train on real negative data, indicating that synthetic negatives may be a method of choice when an appropriate negative dataset is unavailable.

**Figure 8:**
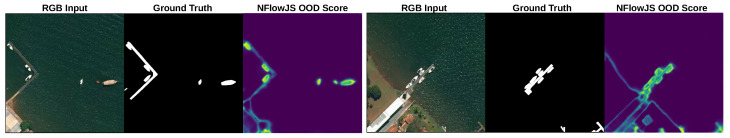
Images from BSB-aerial-OOD (**left columns**). Boats and harbour are selected as OOD samples (**center columns**). NFlowJS delivers accurate OOD scores (**right columns**).

Figure 8 visualizes our performance on the BSB-aerial-OOD dataset. The left column shows the input images. The center column shows OOD objects—harbour and boats. The right column shows that NFlowJS delivers a well-aligned score.

### 5.3. Sensitivity of OOD Detection to Depth

Self-driving applications challenge us to detect anomalies as soon and as far as possible. However, distant anomalies are harder to detect due to being represented with fewer pixels. We analyze the influence of depth to dense OOD detection on the LostAndFound dataset [80]. The LAF test set consists of 1203 images with the corresponding pixel-level disparity maps and calibration parameters of the stereo rig. Due to limitations in the available disparity, we perform analysis in the range from 5 to 50 m. Also, more than 60% of anomalous pixels are closer than 15 m. Hence, the usual metrics (AP and ${\mathrm{FPR}}_{95}$) are biased towards closer ranges. As we further demonstrate, many methods fail to detect anomalies at larger depths. We compare our method with the max-logit (ML) and max-softmax [40] baselines, ODIN [32], SynBoost [19], and OOD head [21]. Table 5 shows that our method produces a low false-positive rate even at high distances. For example, at distances higher than 20 m, we outperform others by a wide margin. This finding is consistent with Figure 6, which shows accurate detection of anomalies at larger distances.

### 5.4. Inference Speed

A convenient dense OOD detector should not drastically increase the already heavy computational burden of semantic segmentation. Hence, we measure the computational overhead of our method and compare it with other approaches. We measure the inference speed on NVIDIA RTX 3090 for 1024×2048 inputs. Table 6 shows that SynBoost [19] and SynthCP [50] are not applicable for real-time inference due to 20× and 3× overhead over our baseline. Our baseline LDN-121 [85] achieves near real-time inference for two megapixel images (46.5 ms, 21.5 FPS). ODIN [32] requires an additional forward–backward pass in order to recover the gradients of the loss with respect to the image. This results in a 3-fold slow-down with respect to the baseline. Similarly, MC Dropout [18] requires K forward passes for prediction with K MC samples. This results in a 45.8 ms overhead when K = 2. NFlowJS increases the inference time for only 7.8 ms with respect to the baseline, while outperforming all previous approaches. The SynthCP measurements are taken from [91], while SynBoost is measured using publicly available code.

### 5.5. Visualization of Synthetic Outliers

**Figure 9:**
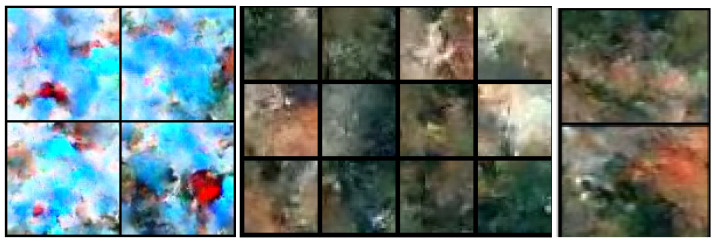
Samples of DenseFlow-25-6 after joint training on aerial images.

Our method is able to generate samples at multiple resolutions with the same normalizing flow. The generated samples have a limited variety when compared to a typical negative dataset, such as ImageNet or COCO [21,51]. However, training with them greatly reduces overconfidence since the model is explicitly trained to produce uncertain predictions in outliers. Figure 9 shows synthetic outliers generated by our normalizing flow after joint training on aerial images. Even though the synthetic negatives look visually abstract, they are a good proxy for real negative data. Consequently, fine-tuning the model on such negatives improves the OOD detection performance.

**Figure 10:**
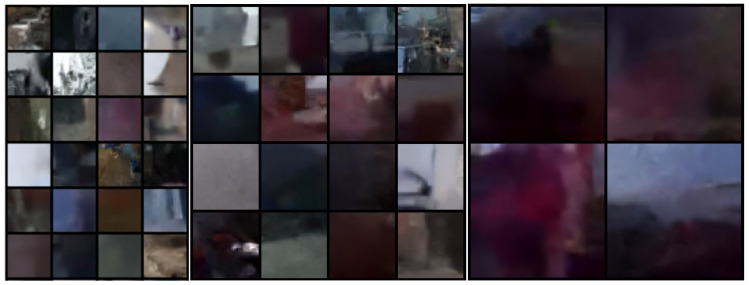
Samples of DenseFlow-25-6 after joint training on road-driving images.

Similarly, Figure 10 shows samples of a normalizing flow after joint training on road-driving scenes. Again, fine-tuning on such negatives improves the OOD detection performance on road-driving scenes. Comparison with Figure 9 reveals that the appearance of our synthetic negative samples strongly depends on the underlying inlier dataset. Samples from Figure 9 resemble lakes and forests, while samples from Figure 10 resemble road, sky, cars, and buildings. These observations do not come as a surprise since our normalizing flows are trained to generate data points along the border of the inlier distribution (cf. Figure 2). In other words, our method reduces the open-space risk of a particular segmentation model by adapting the synthetic negative data to the training dataset.

### 5.6. Synthetic Negatives and Separation in the Feature Space

Up to now, we have considered softmax-activated models. However, softmax can assign arbitrarily large probabilities regardless of the distance from the closest training datum in the feature space [56], which fails to bound open-space risk [52,63]. We analyze the usefulness of synthetic negatives in open-set recognition by considering a popular baseline that is denoted as max-logit [58,59,60]. The max-logit value is proportional to the projection of the feature vector of a given sample onto the closest class prototype vector. This value can be thresholded to bound the open space risk [63,97].

**Figure 11:**
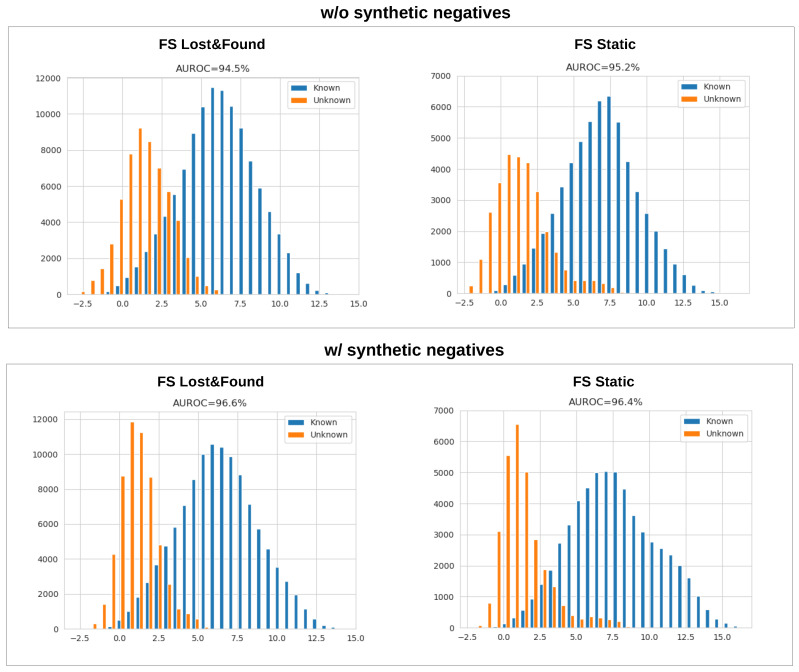
Training on synthetic negative data improves the separation between test inliers and test outliers in the feature space.

The left part of Figure 11 shows histograms of max-logit values for known and unknown pixels on Fishyscapes val. The right part shows the same histograms after fine-tuning with our synthetic negative samples. The figure shows that training with our synthetic negatives increases the separation between known and unknown pixels in feature space and improves the AUROC score. Similar effects have been reported after training with real negative data [61,62]. However, as argued before, our approach avoids the bias towards test anomalies that are related to the training data. Furthermore, it offers a great alternative for non-standard domains, as shown in Table 4. Hence, the proposed method appears to be a promising component of future approaches for dense open-set recognition.

## 6. Ablations

We ablate the impact of loss in negative pixels, the choice of generative model, the impact of pre-training, as well as the impact of temperature scaling on dense OOD detection.

### 6.1. Impacts of the Loss Function and OOD Score

Table 7 analyzes the impact of the loss function $L_{neg}$ and the OOD score $s_{\delta }$ on AnomalyTrack val and ObstacleTrack val. The two chosen datasets feature large and small anomalies, respectively. We separately validate the modulation factor λ for each choice of the negative loss, as well as the temperature parameter. We set T for max-softmax to 10 and for divergence-based scoring functions to 2, which are optimal values. We report the average performance over the last three epochs. Row 1 shows the standard setting with KL divergence as $L_{\mathrm{neg}}$ and max-softmax as the OOD score [26,33]. Row 2 uses the KL divergence both as the loss function and the OOD score. Row 3 features the reverse KL divergence. Minimizing the reverse divergence between the uniform distribution and the softmax distribution is equivalent to maximizing the softmax entropy [51]. Rows 4 and 5 feature the JS divergence loss. The JS divergence substantially outperforms all alternatives both as the loss function (JSD-MSP vs. KL-MSP) and as the OOD score (JSD-JSD vs. JSD-MSP and RKL-RKL). We explain this advantage with a robust response in the synthetic outliers which resemble inliers, as well as with improved consistency during training and scoring (cf. Section 3.2 and Section 3.3).

### 6.2. Impact of the Choice of Generative Model

Table 8 compares synthetic negative data generated by the normalizing flow with synthetic negative data generated by GAN [26] and synthetic negative pre-logit features generated by GMM [44]. Interestingly, training on synthetic OOD features produced by GMM achieves better average precision than synthetic negative images generated by GAN. However, generating synthetic negatives with a normalizing flow outperforms both GAN images and GMM features. This advocates for the advantages of maximum likelihood over adversarial training for the generation of synthetic negatives, as described in Section 3.4. We also note that utilizing RealNVP [27] instead of DenseFlow [75] decreases OOD detection performance.

### 6.3. Impact of Pre-Training

Table 9 explores the impact of pre-training on OOD detection performance. Row 1 shows the performance when neither a generative nor discriminative model are trained prior to the joint training (Section 3.1). In this case, we jointly train both models from their random initializations. Row 2 reveals that discriminative pre-training improves OOD detection. Introducing the synthetic negatives after discriminative pre-training improves generalization. Row 3 shows that pre-training both models generalizes even better.

### 6.4. Impact of Temperature Scaling

Table 10 shows the impact of softmax recalibration on OOD detection. The table explores three different temperatures. We observe that temperature scaling significantly improves the Jensen–Shannon scoring. Values greater than 2 yield worse results.

## 7. Conclusions

We have presented a novel method for dense OOD detection and outlier-aware semantic segmentation. Our method trains on mixed-content images obtained by pasting synthetic negative patches into training images. We produce synthetic negatives by sampling a generative model, which is jointly trained to maximize the likelihood and to give rise to uniform discriminative predictions. Such collaborative learning leads to conservative outlier-aware predictions, which are suitable for OOD detection and outlier-aware semantic segmentation.

We extend the previous work with the following consolidated contributions. First, we replace the adversarial generative model (GAN) with a normalizing flow. We believe that the resulting improvement is due to better coverage of the training distribution. Second, we extend the collaborative training setup for dense prediction. Generative flows are especially well-suited for this task due to straightforward generation at different resolutions. Third, we improve the performance by pre-training the normalizing flow and the discriminative model prior to joint training. Fourth, we propose using the JS divergence as a robust criterion for training a discriminative model with synthetic negatives. We also show that the same criterion can be used as a principled improvement over ad hoc scoring functions, such as max-softmax.

We have evaluated the proposed method on standard benchmarks and datasets for dense OOD detection and outlier-aware segmentation. The results indicate a significant advantage with respect to all previous approaches on the majority of the datasets from two different domains. The advantage becomes substantial in the case of non-standard domains with few suitable auxiliary datasets for sampling real negative data. Additionally, we demonstrate the great potential of our method for real-world deployments due to minimal computational overhead. Suitable avenues for future work include extending our method to setups with bounded open-set risk and other dense prediction tasks.

## Figures and Tables

**Table 1:** Performance evaluation on WD-Pascal [21].

Method	Aux Data	AP ↑	${\mathrm{FPR}}_{95}$↓	AUROC ↑
OOD head [21]	✓	34.9 ± 6.8	40.9 ± 3.9	88.8 ± 1.6
Max-softmax [21]	✓	33.8 ± 5.1	35.5 ± 3.4	91.1 ± 1.0
Void Classifier [12]	✓	25.6 ± 5.5	44.2 ± 4.7	87.7 ± 1.7
MC Dropout [18]	✗	9.7 ± 1.2	41.1 ± 3.7	86.0 ± 1.2
ODIN [32]	✗	6.0 ± 0.5	53.7 ± 7.0	79.9 ± 1.5
Max-softmax [40]	✗	5.0 ± 0.5	48.8 ± 4.7	78.7 ± 1.5
NFlowJS (ours)	✗	30.2 ± 4.1	32.3 ± 5.9	92.3 ± 1.3

**Table 3:** Performance evaluation on StreetHazards [59].

Method	Aux.	Anomaly Det.		Closed	Open	
	Data	AP	${\mathrm{FPR}}_{95}$	$\bar{\mathrm{IoU}}$	$\bar{\mathrm{IoU}}$	o- $\bar{\mathrm{IoU}}$
SynthCP [50]	✗	9.3	28.4	-	-	-
TRADI [94]	✗	7.2	25.3	-	-	-
OVNNI [95]	✗	12.6	22.2	54.6	-	-
Synthetic Outliers + Entropy [28]	✗	12.7	25.2	59.7	-	-
Deep Metric Learning [57]	✗	14.7	17.3	-	-	-
Max-softmax [40]	✗	7.5	27.9	65.0	32.4	35.1
Max logit [59]	✗	11.6	22.5	65.0	38.0	41.2
ODIN [32]	✗	7.0	28.7	65.0	-	28.8
ReAct [96]	✗	10.9	21.2	62.7	31.8	34.0
Energy [93]	✓	12.9	18.2	63.3	39.6	42.7
Max-softmax + Outlier Exposure [33]	✓	14.6	17.7	61.7	40.8	43.8
Outlier Head [21]	✓	19.7	56.2	66.6	-	33.9
Outlier Head*Max Softmax [13]	✓	18.8	30.9	66.6	-	43.6
NFlowJS (ours)	✗	22.2	16.2	65.0	41.6	44.9

**Table 4:** Performance evaluation on images from BSB-aerial-OOD.

Method	Aux. Data	AP	${\mathrm{FPR}}_{95}$	AUROC
Max-softmax [40]	✗	35.1	13.5	96.4
Max-softmax + Outlier Exposure [33]	✓	32.2	9.6	97.0
Energy [93]	✓	38.1	11.0	96.8
GAN negatives [26]	✗	31.7	9.2	96.9
MOP [17]	✗	24.5	10.9	96.0
Dirichlet Prior Network [25]	✓	27.3	9.1	97.1
JSDiv (ours)	✓	38.4	12.5	96.5
NFlowJS (ours)	✗	44.1	8.8	97.8

**Table 5:** Analysis of ${\mathrm{FPR}}_{95}$ at various distances from the camera.

Range (in Meters)	NFlowJS	MSP	ML	SynBoost	OOD-Head	ODIN
5–10	0.7	16.6	4.7	0.2	7.9	10.9
10–15	1.2	18.0	7.3	17.7	10.6	9.0
15–20	0.8	19.3	5.9	25.0	16.9	11.1
20–25	1.1	23.2	5.8	23.3	23.6	13.4
25–30	1.8	28.0	7.1	18.8	26.7	16.6
30–35	2.7	32.6	7.6	27.4	30.8	22.6
35–40	3.5	37.9	10.1	25.4	36.8	25.9
40–45	5.6	41.4	13.2	25.8	42.2	30.3
45–50	8.8	46.3	15.8	29.9	52.0	37.9

**Table 6:** Comparison of inference speed on 2MPix images and RTX3090.

Method	Resynth.	Infer. Time (ms)	FPS
SynthCP [50]	✓	146.9	6.8
SynBoost [19]	✓	1055.5	<1
LDN-121 (Base) [85]	✗	46.5	21.5
Base + ODIN [32]	✗	+149.1	5.11
Base + MC = 2 Dropout [18]	✗	+45.8	10.83
Base + NFlowJS (ours)	✗	+7.8	18.4

**Table 7:** Validation of the loss in negative pixels and the OOD score.

Loss	s(x)	AnomalyTrack-val		ObstacleTrack-val	
		AP	${\mathrm{FPR}}_{95}$	AP	${\mathrm{FPR}}_{95}$
KL	MSP	57.5 ± 0.7	29.0 ± 1.7	95.1 ± 0.2	0.2 ± 0.1
KL	KL	55.7 ± 0.4	26.3 ± 1.3	94.3 ± 0.2	0.1 ± 0.0
RKL	RKL	57.0 ± 0.3	28.9 ± 0.3	94.4 ± 0.1	0.3 ± 0.0
JSD	MSP	63.0 ± 0.5	22.8 ± 0.7	96.1 ± 0.2	0.2 ± 0.0
JSD	JSD	63.3 ± 0.6	19.8 ± 0.8	95.8 ± 0.2	0.1 ± 0.0

**Table 8:** Impact of generative model on OOD detection performance.

Generator	AnomalyTrack-val		ObstacleTrack-val	
	AP	${\mathrm{FPR}}_{95}$	AP	${\mathrm{FPR}}_{95}$
GMM-VOS	56.7 ± 0.2	28.0 ± 0.4	81.8 ± 0.5	3.9 ± 0.2
GAN	56.1 ± 0.4	26.1 ± 0.6	80.8 ± 0.4	3.6 ± 0.1
NFlow	61.4 ± 0.8	21.7 ± 1.3	94.9 ± 0.1	0.1 ± 0.1

**Table 9:** Impact of pre-training on the success of joint training.

Cls.	Flow	AnomalyTrack-val		ObstacleTrack-val	
		AP	${\mathrm{FPR}}_{95}$	AP	${\mathrm{FPR}}_{95}$
✗	✗	56.9 ± 1.2	27.8 ± 2.1	90.5 ± 0.3	1.0 ± 0.1
✓	✗	61.4 ± 0.8	21.7 ± 1.3	94.9 ± 0.1	0.1 ± 0.1
✓	✓	63.3 ± 0.6	19.8 ± 0.8	95.8 ± 0.2	0.1 ± 0.0

**Table 10:** Impact of temperature on JSD scoring.

Temp.	AnomalyTrack-val		ObstacleTrack-val	
	AP	${\mathrm{FPR}}_{95}$	AP	${\mathrm{FPR}}_{95}$
T = 1	59.7 ± 0.5	40.0 ± 0.8	92.6 ± 0.3	1.1 ± 0.1
T = 1.5	62.7 ± 0.6	23.7 ± 0.9	95.3 ± 0.2	0.2 ± 0.0
T = 2	63.3 ± 0.6	19.8 ± 0.8	95.8 ± 0.2	0.1 ± 0.0

## Data Availability

Data are contained within the article.

## References

[1] Everingham M., Gool L.V., Williams C.K.I., Winn J.M., Zisserman A. (2010). The Pascal Visual Object Classes (VOC) Challenge. Int. J. Comput. Vis..

[2] Farabet C., Couprie C., Najman L., LeCun Y. (2013). Learning Hierarchical Features for Scene Labeling. IEEE Trans. Pattern Anal. Mach. Intell..

[3] Cheng B., Collins M.D., Zhu Y., Liu T., Huang T.S., Adam H., Chen L. Panoptic-DeepLab: A Simple, Strong, and Fast Baseline for Bottom-Up Panoptic Segmentation. Proceedings of the 2020 IEEE/CVF Conference on Computer Vision and Pattern Recognition.

[4] Godard C., Aodha O.M., Firman M., Brostow G.J. Digging Into Self-Supervised Monocular Depth Estimation. Proceedings of the 2019 IEEE/CVF International Conference on Computer Vision.

[5] Luc P., Couprie C., LeCun Y., Verbeek J. Predicting Future Instance Segmentation by Forecasting Convolutional Features. Proceedings of the 15th European Conference on Computer Vision, ECCV.

[6] Uhlemeyer S., Rottmann M., Gottschalk H. Towards Unsupervised Open World Semantic Segmentation. Proceedings of the the 38th Conference on Uncertainty in Artificial Intelligence.

[7] Orsic M., Segvic S. (2021). Efficient semantic segmentation with pyramidal fusion. Pattern Recognit..

[8] Guo C., Pleiss G., Sun Y., Weinberger K.Q. On Calibration of Modern Neural Networks. Proceedings of the 34th International Conference on Machine Learning, ICML.

[9] Zendel O., Honauer K., Murschitz M., Steininger D., Dominguez G.F. WildDash—Creating Hazard-Aware Benchmarks. Proceedings of the European Conference on Computer Vision (ECCV).

[10] Sakaridis C., Dai D., Van Gool L. ACDC: The Adverse Conditions Dataset with Correspondences for Semantic Driving Scene Understanding. Proceedings of the IEEE/CVF International Conference on Computer Vision (ICCV).

[11] Chan R., Lis K., Uhlemeyer S., Blum H., Honari S., Siegwart R., Salzmann M., Fua P., Rottmann M. (2021). SegmentMeIfYouCan: A Benchmark for Anomaly Segmentation. arXiv.

[12] Blum H., Sarlin P.E., Nieto J., Siegwart R., Cadena C. (2021). The Fishyscapes Benchmark: Measuring Blind Spots in Semantic Segmentation. Int. J. Comput. Vis..

[13] Bevandić P., Krešo I., Oršić M., Šegvić S. (2022). Dense open-set recognition based on training with noisy negative images. Image Vis. Comput..

[14] Lis K., Nakka K.K., Fua P., Salzmann M. Detecting the Unexpected via Image Resynthesis. Proceedings of the International Conference on Computer Vision, ICCV.

[15] Vojir T., Šipka T., Aljundi R., Chumerin N., Reino D.O., Matas J. Road Anomaly Detection by Partial Image Reconstruction With Segmentation Coupling. Proceedings of the International Conference on Computer Vision, ICCV.

[16] De Carvalho O.L.F., de Carvalho Júnior O.A., Silva C.R., de Albuquerque A.O., Santana N.C., Borges D.L., Gomes R.A.T., Guimarães R.F. (2022). Panoptic Segmentation Meets Remote Sensing. Remote Sens..

[17] Da Silva C.C.V., Nogueira K., Oliveira H.N., dos Santos J.A. Towards Open-Set Semantic Segmentation of Aerial Images. Proceedings of the 2020 IEEE Latin American GRSS & ISPRS Remote Sensing Conference (LAGIRS).

[18] Kendall A., Gal Y. What Uncertainties Do We Need in Bayesian Deep Learning for Computer Vision?. Proceedings of the Neural Information Processing Systems.

[19] Biase G.D., Blum H., Siegwart R., Cadena C. Pixel-Wise Anomaly Detection in Complex Driving Scenes. Proceedings of the Computer Vision and Pattern Recognition, CVPR.

[20] Lis K., Honari S., Fua P., Salzmann M. (2020). Detecting Road Obstacles by Erasing Them. IEEE Trans. Pattern Anal. Mach. Intell..

[21] Bevandic P., Kreso I., Orsic M., Segvic S. Simultaneous Semantic Segmentation and Outlier Detection in Presence of Domain Shift. Proceedings of the 41st DAGM German Conference, DAGM GCPR.

[22] van Amersfoort J., Smith L., Jesson A., Key O., Gal Y. (2021). On Feature Collapse and Deep Kernel Learning for Single Forward Pass Uncertainty. arXiv.

[23] Perera P., Morariu V.I., Jain R., Manjunatha V., Wigington C., Ordonez V., Patel V.M. Generative-Discriminative Feature Representations for Open-Set Recognition. Proceedings of the Computer Vision and Pattern Recognition, CVPR.

[24] González C., Gotkowski K., Fuchs M., Bucher A., Dadras A., Fischbach R., Kaltenborn I.J., Mukhopadhyay A. (2022). Distance-based detection of out-of-distribution silent failures for COVID-19 lung lesion segmentation. Med. Image Anal..

[25] Gawlikowski J., Saha S., Kruspe A.M., Zhu X.X. Towards Out-of-Distribution Detection for Remote Sensing. Proceedings of the IEEE International Geoscience and Remote Sensing Symposium, IGARSS.

[26] Lee K., Lee H., Lee K., Shin J. Training Confidence-calibrated Classifiers for Detecting Out-of-Distribution Samples. Proceedings of the 6th International Conference on Learning Representations, ICLR.

[27] Dinh L., Sohl-Dickstein J., Bengio S. Density estimation using Real NVP. Proceedings of the 5th International Conference on Learning Representations, ICLR.

[28] Grcić M., Bevandić P., Šegvić S. Dense Open-set Recognition with Synthetic Outliers Generated by Real NVP. Proceedings of the 16th International Joint Conference on Computer Vision, Imaging and Computer Graphics Theory and Applications, VISIGRAPP.

[29] Hawkins D.M. (1980). Identification of Outliers.

[30] Ruff L., Kauffmann J.R., Vandermeulen R.A., Montavon G., Samek W., Kloft M., Dietterich T.G., Müller K. (2021). A Unifying Review of Deep and Shallow Anomaly Detection. Proc. IEEE.

[31] Zhang L.H., Goldstein M., Ranganath R. Understanding Failures in Out-of-Distribution Detection with Deep Generative Models. Proceedings of the 38th International Conference on Machine Learning, ICML.

[32] Liang S., Li Y., Srikant R. Enhancing The Reliability of Out-of-distribution Image Detection in Neural Networks. Proceedings of the 6th International Conference on Learning Representations, ICLR.

[33] Hendrycks D., Mazeika M., Dietterich T.G. Deep Anomaly Detection with Outlier Exposure. Proceedings of the 7th International Conference on Learning Representations, ICLR.

[34] Zhou K., Li J., Xiao Y., Yang J., Cheng J., Liu W., Luo W., Liu J., Gao S. (2022). Memorizing Structure-Texture Correspondence for Image Anomaly Detection. IEEE Trans. Neural Netw. Learn. Syst..

[35] Yang Z., Zhang T., Bozchalooi I.S., Darve E. (2022). Memory-Augmented Generative Adversarial Networks for Anomaly Detection. IEEE Trans. Neural Netw. Learn. Syst..

[36] Li W., Mahadevan V., Vasconcelos N. (2014). Anomaly Detection and Localization in Crowded Scenes. IEEE Trans. Pattern Anal. Mach. Intell..

[37] Bergmann P., Batzner K., Fauser M., Sattlegger D., Steger C. (2021). The MVTec Anomaly Detection Dataset: A Comprehensive Real-World Dataset for Unsupervised Anomaly Detection. Int. J. Comput. Vis..

[38] Wang X., Che Z., Jiang B., Xiao N., Yang K., Tang J., Ye J., Wang J., Qi Q. (2022). Robust Unsupervised Video Anomaly Detection by Multipath Frame Prediction. IEEE Trans. Neural Netw. Learn. Syst..

[39] Massoli F.V., Falchi F., Kantarci A., Akti S., Ekenel H.K., Amato G. (2022). MOCCA: Multilayer One-Class Classification for Anomaly Detection. IEEE Trans. Neural Netw. Learn. Syst..

[40] Hendrycks D., Gimpel K. A Baseline for Detecting Misclassified and Out-of-Distribution Examples in Neural Networks. Proceedings of the 5th International Conference on Learning Representations, ICLR.

[41] Macêdo D., Ren T.I., Zanchettin C., Oliveira A.L.I., Ludermir T.B. (2022). Entropic Out-of-Distribution Detection: Seamless Detection of Unknown Examples. IEEE Trans. Neural Netw. Learn. Syst..

[42] Dhamija A.R., Günther M., Boult T.E. Reducing Network Agnostophobia. Proceedings of the Annual Conference on Neural Information Processing Systems 2018, NeurIPS 2018.

[43] Lucas T., Shmelkov K., Alahari K., Schmid C., Verbeek J. Adaptive Density Estimation for Generative Models. Proceedings of the Neural Information Processing Systems.

[44] Du X., Wang Z., Cai M., Li Y. VOS: Learning What You Don’t Know by Virtual Outlier Synthesis. Proceedings of the Tenth International Conference on Learning Representations, ICLR 2022.

[45] Zhao Z., Cao L., Lin K. (2023). Revealing the Distributional Vulnerability of Discriminators by Implicit Generators. IEEE Trans. Pattern Anal. Mach. Intell..

[46] Kumar N., Segvic S., Eslami A., Gumhold S. Normalizing Flow based Feature Synthesis for Outlier-Aware Object Detection. Proceedings of the IEEE/CVF Conference on Computer Vision and Pattern Recognition, CVPR.

[47] Blum H., Sarlin P., Nieto J.I., Siegwart R., Cadena C. Fishyscapes: A Benchmark for Safe Semantic Segmentation in Autonomous Driving. Proceedings of the IEEE/CVF International Conference on Computer Vision Workshops.

[48] Du X., Wang X., Gozum G., Li Y. Unknown-Aware Object Detection: Learning What You Don’t Know from Videos in the Wild. Proceedings of the IEEE/CVF Conference on Computer Vision and Pattern Recognition, CVPR 2022.

[49] Riedlinger T., Rottmann M., Schubert M., Gottschalk H. Gradient-Based Quantification of Epistemic Uncertainty for Deep Object Detectors. Proceedings of the IEEE/CVF Winter Conference on Applications of Computer Vision, WACV 2023.

[50] Xia Y., Zhang Y., Liu F., Shen W., Yuille A.L. Synthesize Then Compare: Detecting Failures and Anomalies for Semantic Segmentation. Proceedings of the 16th European Conference on Computer Vision, ECCV.

[51] Chan R., Rottmann M., Gottschalk H. Entropy Maximization and Meta Classification for Out-of-Distribution Detection in Semantic Segmentation. Proceedings of the International Conference on Computer Vision, ICCV.

[52] Scheirer W.J., de Rezende Rocha A., Sapkota A., Boult T.E. (2013). Toward Open Set Recognition. IEEE Trans. Pattern Anal. Mach. Intell..

[53] Bendale A., Boult T.E. Towards Open Set Deep Networks. Proceedings of the IEEE Conference on Computer Vision and Pattern Recognition, CVPR.

[54] Zhang H., Li A., Guo J., Guo Y. Hybrid Models for Open Set Recognition. Proceedings of the 16th European Conference on Computer Vision ECCV.

[55] Oliveira H., Silva C., Machado G.L.S., Nogueira K., dos Santos J.A. (2021). Fully convolutional open set segmentation. Mach. Learn..

[56] Scheirer W.J., Jain L.P., Boult T.E. (2014). Probability Models for Open Set Recognition. IEEE Trans. Pattern Anal. Mach. Intell..

[57] Cen J., Yun P., Cai J., Wang M.Y., Liu M. Deep Metric Learning for Open World Semantic Segmentation. Proceedings of the IEEE/CVF International Conference on Computer Vision.

[58] Chen G., Peng P., Wang X., Tian Y. (2022). Adversarial Reciprocal Points Learning for Open Set Recognition. IEEE Trans. Pattern Anal. Mach. Intell..

[59] Hendrycks D., Basart S., Mazeika M., Mostajabi M., Steinhardt J., Song D. (2019). Scaling out-of-distribution detection for real-world settings. arXiv.

[60] Vaze S., Han K., Vedaldi A., Zisserman A. Open-Set Recognition: A Good Closed-Set Classifier is All You Need. Proceedings of the the Tenth International Conference on Learning Representations, ICLR 2022.

[61] Neal L., Olson M.L., Fern X.Z., Wong W., Li F. Open Set Learning with Counterfactual Images. Proceedings of the European Conference on Computer Vision.

[62] Kong S., Ramanan D. OpenGAN: Open-Set Recognition Via Open Data Generation. Proceedings of the 2021 IEEE/CVF International Conference on Computer Vision (ICCV).

[63] Boult T.E., Cruz S., Dhamija A.R., Günther M., Henrydoss J., Scheirer W.J. Learning and the Unknown: Surveying Steps toward Open World Recognition. Proceedings of the AAAI Conference on Artificial Intelligence.

[64] Geng C., Huang S., Chen S. (2021). Recent Advances in Open Set Recognition: A Survey. IEEE Trans. Pattern Anal. Mach. Intell..

[65] Brilhadoz A., Gutoski M., Lazzaretti A.E., Lopes H.S. A Comparative Study for Open Set Semantic Segmentation Methods. Proceedings of the XV Congresso Brasileiro de Inteligência Computacional.

[66] Michieli U., Zanuttigh P. (2021). Knowledge distillation for incremental learning in semantic segmentation. Comput. Vis. Image Underst..

[67] Yu Y., Ji Z., Guo J., Pang Y. (2018). Transductive Zero-Shot Learning with Adaptive Structural Embedding. IEEE Trans. Neural Netw. Learn. Syst..

[68] Shaban A., Bansal S., Liu Z., Essa I., Boots B. One-Shot Learning for Semantic Segmentation. Proceedings of the British Machine Vision Conference, BMVC.

[69] Lu J., Jin S., Liang J., Zhang C. (2021). Robust Few-Shot Learning for User-Provided Data. IEEE Trans. Neural Netw. Learn. Syst..

[70] Salakhutdinov R., Hinton G. Deep Boltzmann Machines. Proceedings of the Twelth International Conference on Artificial Intelligence and Statistics.

[71] Van Oord A., Kalchbrenner N., Kavukcuoglu K. Pixel recurrent neural networks. Proceedings of the International Conference on Machine Learning.

[72] Kingma D.P., Welling M. Auto-Encoding Variational Bayes. Proceedings of the International Conference on Learning Representations, ICLR.

[73] Vahdat A., Kautz J. NVAE: A Deep Hierarchical Variational Autoencoder. Proceedings of the Neural Information Processing Systems.

[74] Goodfellow I.J., Pouget-Abadie J., Mirza M., Xu B., Warde-Farley D., Ozair S., Courville A.C., Bengio Y. (2020). Generative adversarial networks. Commun. ACM.

[75] Grcić M., Grubišić I., Šegvić S. Densely connected normalizing flows. Proceedings of the Neural Information Processing Systems.

[76] Kingma D.P., Dhariwal P. Glow: Generative Flow with Invertible 1 × 1 Convolutions. Proceedings of the Neural Information Processing Systems.

[77] Metz L., Poole B., Pfau D., Sohl-Dickstein J. Unrolled Generative Adversarial Networks. Proceedings of the 5th International Conference on Learning Representations.

[78] Maag K., Chan R., Uhlemeyer S., Kowol K., Gottschalk H. Two Video Data Sets for Tracking and Retrieval of Out of Distribution Objects. Proceedings of the Asian Conference on Computer Vision.

[79] Bevandic P., Kreso I., Orsic M., Segvic S. (2018). Discriminative out-of-distribution detection for semantic segmentation. arXiv.

[80] Pinggera P., Ramos S., Gehrig S., Franke U., Rother C., Mester R. Lost and Found: Detecting small road hazards for self-driving vehicles. Proceedings of the International Conference on Intelligent Robots and Systems, IROS.

[81] Grcic M., Bevandic P., Segvic S. DenseHybrid: Hybrid Anomaly Detection for Dense Open-set Recognition. Proceedings of the European Conference on Computer Vision.

[82] Vaze S., Han K., Vedaldi A., Zisserman A. The Semantic Shift Benchmark. Proceedings of the ICML 2022 Shift Happens Workshop.

[83] Zhu X.X., Hu J., Qiu C., Shi Y., Kang J., Mou L., Bagheri H., Häberle M., Hua Y., Huang R. (2019). So2Sat LCZ42: A Benchmark Dataset for Global Local Climate Zones Classification. arXiv.

[84] Davis J., Goadrich M. The relationship between Precision-Recall and ROC curves. Proceedings of the International Conference on Machine Learning (ICML 2006).

[85] Kreso I., Krapac J., Segvic S. (2021). Efficient Ladder-Style DenseNets for Semantic Segmentation of Large Images. IEEE Trans. Intell. Transp. Syst..

[86] Cordts M., Omran M., Ramos S., Rehfeld T., Enzweiler M., Benenson R., Franke U., Roth S., Schiele B. The Cityscapes Dataset for Semantic Urban Scene Understanding. Proceedings of the IEEE Conference on Computer Vision and Pattern Recognition, CVPR.

[87] Neuhold G., Ollmann T., Bulò S.R., Kontschieder P. The Mapillary Vistas Dataset for Semantic Understanding of Street Scenes. Proceedings of the IEEE International Conference on Computer Vision, ICCV.

[88] Bulò S.R., Porzi L., Kontschieder P. In-Place Activated BatchNorm for Memory-Optimized Training of DNNs. Proceedings of the Conference on Computer Vision and Pattern Recognition.

[89] Barron J.T. A General and Adaptive Robust Loss Function. Proceedings of the IEEE Conference on Computer Vision and Pattern Recognition.

[90] Zhu Y., Sapra K., Reda F.A., Shih K.J., Newsam S.D., Tao A., Catanzaro B. Improving Semantic Segmentation via Video Propagation and Label Relaxation. Proceedings of the Conference on Computer Vision and Pattern Recognition, CVPR.

[91] Jung S., Lee J., Gwak D., Choi S., Choo J. Standardized Max Logits: A Simple yet Effective Approach for Identifying Unexpected Road Obstacles in Urban-Scene Segmentation. Proceedings of the International Conference on Computer Vision, ICCV.

[92] Malinin A., Gales M.J.F. Predictive Uncertainty Estimation via Prior Networks. Proceedings of the Annual Conference on Neural Information Processing Systems.

[93] Liu W., Wang X., Owens J.D., Li Y. Energy-based Out-of-distribution Detection. Proceedings of the Thirty-Fourth Annual Conference on Neural Information Processing Systems NeurIPS.

[94] Franchi G., Bursuc A., Aldea E., Dubuisson S., Bloch I. TRADI: Tracking Deep Neural Network Weight Distributions. Proceedings of the 16th European Conference on Computer Vision, ECCV.

[95] Franchi G., Bursuc A., Aldea E., Dubuisson S., Bloch I. (2020). One Versus all for deep Neural Network Incertitude (OVNNI) quantification. arXiv.

[96] Sun Y., Guo C., Li Y. ReAct: Out-of-distribution Detection With Rectified Activations. Proceedings of the Thirty-Fifth Annual Conference on Neural Information Processing Systems NeurIPS.

[97] Stefano C.D., Sansone C., Vento M. (2000). To reject or not to reject: That is the question-an answer in case of neural classifiers. IEEE Trans. Syst. Man Cybern. Part C.

